# Phylogenetic analysis and classification of the *Brassica rapa *SET-domain protein family

**DOI:** 10.1186/1471-2229-11-175

**Published:** 2011-12-14

**Authors:** Yong Huang, Chunlin Liu, Wen-Hui Shen, Ying Ruan

**Affiliations:** 1 Hunan Provincial Key Laboratory of Crop Germplasm Innovation and Utilization, Hunan Agricultural University, Changsha 410128, China; 2 College of Bioscience and Biotechnology, Hunan Agricultural University, Changsha 410128, China; 3 Institut de Biologie Moléculaire des Plantes du CNRS, Université de Strasbourg, 12 rue du Général Zimmer, 67084 Strasbourg Cedex, France

**Keywords:** Chromatin, Histone, Lysine methylation, SET domain, Gene duplication, Nomenclature

## Abstract

**Background:**

The SET (*Su(var)3-9, Enhancer-of-zeste, Trithorax*) domain is an evolutionarily conserved sequence of approximately 130-150 amino acids, and constitutes the catalytic site of lysine methyltransferases (KMTs). KMTs perform many crucial biological functions *via *histone methylation of chromatin. Histone methylation marks are interpreted differently depending on the histone type (i.e. H3 or H4), the lysine position (e.g. H3K4, H3K9, H3K27, H3K36 or H4K20) and the number of added methyl groups (i.e. me1, me2 or me3). For example, H3K4me3 and H3K36me3 are associated with transcriptional activation, but H3K9me2 and H3K27me3 are associated with gene silencing. The substrate specificity and activity of KMTs are determined by sequences within the SET domain and other regions of the protein.

**Results:**

Here we identified 49 SET-domain proteins from the recently sequenced *Brassica rapa *genome. We performed sequence similarity and protein domain organization analysis of these proteins, along with the SET-domain proteins from the dicot *Arabidopsis thaliana*, the monocots *Oryza sativa *and *Brachypodium distachyon*, and the green alga *Ostreococcus tauri. *We showed that plant SET-domain proteins can be grouped into 6 distinct classes, namely KMT1, KMT2, KMT3, KMT6, KMT7 and S-ET. Apart from the S-ET class, which has an interrupted SET domain and may be involved in methylation of nonhistone proteins, the other classes have characteristics of histone methyltransferases exhibiting different substrate specificities: KMT1 for H3K9, KMT2 for H3K4, KMT3 for H3K36, KMT6 for H3K27 and KMT7 also for H3K4. We also propose a coherent and rational nomenclature for plant SET-domain proteins. Comparisons of sequence similarity and synteny of *B. rapa *and *A. thaliana *SET-domain proteins revealed recent gene duplication events for some KMTs.

**Conclusion:**

This study provides the first characterization of the SET-domain KMT proteins of *B. rapa*. Phylogenetic analysis data allowed the development of a coherent and rational nomenclature of this important family of proteins in plants, as in animals. The results obtained in this study will provide a base for nomenclature of KMTs in other plant species and facilitate the functional characterization of these important epigenetic regulatory genes in *Brassica *crops.

## Background

Epigenetic regulation acts through heritable changes in genome function that occur without a change in DNA sequence. One well-known epigenetic mechanism is through posttranslational covalent modifications of histones; these modifications include acetylation, methylation, ubiquitylation and others, and form the basis of the 'histone code' for gene regulation [1]. Histone lysine methylation plays a pivotal role in a wide range of cellular processes including heterochromatin formation, transcriptional regulation, parental imprinting, and cell fate determination [2]. At least six lysine residues, five on histone H3 (K4, K9, K27, K36, K79) and one on H4 (K20), are subject to methylation. Each lysine can carry one, two or three methyl residue(s), known as mono-, di- and tri-methylation, respectively. In general, di-/tri- methylation of H3K4 and H3K36 correlates with transcriptional activation, whereas di-methylation of H3K9 and trimethylation of H3K27 correlates with gene silencing in plants and animals [2,3].

All known lysine methylation modifications, with the exception of H3K79 methylation, are carried out by methyltransferases that contain an evolutionarily conserved SET domain, named after three *Drosophila *genes (*Su(var)*, *E(z)*, and *Trithorax*) [4]. The SET domain encompasses approximately 130-150 amino acids that form a knot-like structure and constitute the enzyme catalytic site for lysine methylation [5]. In addition to the SET domain, flanking sequences, more distant protein domains, and possibly some cofactors are also important for enzyme activity and specificity. The genes encoding SET-domain proteins are ancient, existing in prokaryotes and eukaryotes, but have proliferated and evolved novel functions connected with the appearance of eukaryotes [6].

The first plant genes encoding SET-domain proteins to be genetically characterized were *CURLY LEAF *(*CLF*) and *MEDEA *(*MEA*) in *Arabidopsis thaliana *[7,8]. Chromatin-binding properties and histone methylation activity of plant SET-domain proteins were first reported for tobacco NtSET1 and *Arabidopsis *KRYPTONITE (KYP) [9,10]. Phylogenetic analysis of plant SET domain proteins has proven helpful as a guide for genetic and molecular studies of this large family of proteins [11,12]. To date, some of the *Arabidopsis *SET-domain family members have been characterized and shown to play crucial functions in diverse processes including flowering time control, cell fate determination, leaf morphogenesis, floral organogenesis, parental imprinting and seed development [3,13-15].

Genome sequences of an increasing number of plant species, in addition to the model plants (*Arabidopsis thaliana*, *Oryza sativa*, and *Brachypodium distachyon*), have also been completed. Other *Brassica *species are of particular interest because of their agro-economical importance and their close relationship with *Arabidopsis*, thus providing insights into recent SET-domain gene amplification during evolution of *Brassica *species. Here, we identified and analyzed 49 SET-domain proteins from the recently completed *Brassica rapa *whole genome sequence [16]. Our data provide a platform for future functional characterization of these important epigenetic regulatory genes in *Brassica *species.

## Results

### Identification of SET-domain proteins from the *B. rapa *genome

Using BLASTp and tBLASTn with the full complement of known *Arabidopsis *and rice SET-domain proteins as queries, we identified 49 genes encoding different SET-domain proteins from the *B. rapa *genome (http://brassicadb.org/brad). We used the nomenclature recently proposed for lysine methyltransferases (KMTs, [17]) and named the newly identified *B. rapa *genes based on our phylogenetic analysis of their corresponding protein sequences (see below). Apart from *BrKMT1B;1a *and *BrKMT1B;2b *genes, whose chromosomal locations are yet unknown, the other 47 genes are distributed on the ten *B. rapa *chromosomes, with 1-7 *KMT *genes per chromosome (Table 1).

**Table 1:** List of green lingeage SET-domain proteins analyzed in this study

*B. rapa*			*A. thaliana*		*O. sativa*		*B. distachyon*		*O. tauri*	
**Name**	**Locus**	**Chr**.	**Name**	**Synonyms**	**Name**	**Synonyms**	**Name**	**Locus**	**Name**	**Synonyms**

BrKMT1A;1	Bra006226	3	AtKMT1A;1	SDG33/SUVH4/KYP	OsKMT1A;1	SDG714	BdKMT1A;1a	Bradi2g59430		

BrKMT1A;2a	Bra021840	4	AtKMT1A;2a	SDG3/SUVH2	OsKMT1A;2a	SDG726	BdKMT1A;1b	Bradi2g26840		

BrKMT1A;2b	Bra007048	3	AtKMT1A;2b	SDG22/SUVH9	OsKMT1A;2b	SDG715	BdKMT1A;2a	Bradi1g29010		

BrKMT1A;2c	Bra005511	5	AtKMT1A;3a	SDG9/SUVH5	OsKMT1A;3a	SDG710	BdKMT1A;2b	Bradi3g12710		

BrKMT1A;3a	Bra005362	5	AtKMT1A;3b	SDG23/SUVH6	OsKMT1A;3b	SDG727	BdKMT1A;2c	Bradi2g60080		

BrKMT1A;3b	Bra030212	4	AtKMT1A;4a	SDG32/SUVH1	OsKMT1A;3c	SDG703	BdKMT1A;3a	Bradi3g35330		

BrKMT1A;4a	Bra009408	10	AtKMT1A;4b	SDG19/SUVH3	OsKMT1A;4a	SDG704	BdKMT1A;3b	Bradi4g28460		

BrKMT1A;4b	Bra016018	7	AtKMT1A;4c	SDG17/SUVH7	OsKMT1A;4b	SDG713	BdKMT1A;3c	Bradi4g28792		

BrKMT1A;4c	Bra028776	2	AtKMT1A;4d	SDG21/SUVH8	OsKMT1A;4c	SDG728	BdKMT1A;3d	Bradi5g17340		

BrKMT1A;4d	Bra005829	3	AtKMT1A;4e	SDG11/SUVH10	OsKMT1A;4d	SDG709	BdKMT1A;4a	Bradi1g53840		

BrKMT1A;4e	Bra004258	7			OsKMT1A;4e	SDG734	BdKMT1A;4b	Bradi4g25940		

BrKMT1A;4f	Bra025949	6			OsKMT1A;4f	SDG733	BdKMT1A;4c	Bradi2g13416		

BrKMT1A;4g	Bra009409	10					BdKMT1A;4d	Bradi4g43717		

BrKMT1A;4h	Bra025950	6								

BrKMT1B;1a	Bra040197	ud*	AtKMT1B;1	SDG31/SUVR4	OsKMT1B;1	SDG742	BdKMT1B;1a	Bradi3g12790		

BrKMT1B;1b	Bra001104	3	AtKMT1B;2a	SDG13/SUVR1	OsKMT1B;2	SDG712	BdKMT1B;1b	Bradi5g00500		

BrKMT1B;2a	Bra033710	6	AtKMT1B;2b	SDG18/SUVR2	OsKMT1B;3	SDG706	BdKMT1B;2a	Bradi1g64870		

BrKMT1B;2b	Bra036004	ud*	AtKMT1B;3	SDG6/SUVR5/AtCZS	OsKMT1B;4	SDG729	BdKMT1B;2b	Bradi3g48970		

BrKMT1B;3	Bra032148	4	AtKMT1B;4	SDG20/SUVR3			BdKMT1B;2c	Bradi3g48980		

BrKMT1B;4	Bra031976	2					BdKMT1B;3	Bradi3g52950		

							BdKMT1B;4	Bradi2g51320		

BrKMT2;1a	Bra021721	4	AtKMT2;1a	SDG27/ATX1	OsKMT2;1	SDG723	BdKMT2;1	Bradi4g08510	OtKMT2	SDG3601

BrKMT2;1b	Bra032450	9	AtKMT2;1b	SDG30/ATX2	OsKMT2;2a	SDG717	BdKMT2;2a	Bradi4g01790		

BrKMT2;2	Bra027983	9	AtKMT2;2	SDG25/ATXR7	OsKMT2;2b	SDG732	BdKMT2;2b	Bradi3g13430		

BrKMT2;3a	Bra003467	7	AtKMT2;3a	SDG14/ATX3	OsKMT2;3a	SDG721	BdKMT2;2c	Bradi4g37627		

BrKMT2;3b	Bra040838	8	AtKMT2;3b	SDG16/ATX4	OsKMT2;3b	SDG705	BdKMT2;3a	Bradi2g07082		

BrKMT2;3c	Bra003056	10	AtKMT2;3c	SDG29/ATX5			BdKMT2;3b	Bradi2g45430		

BrKMT3;1	Bra015678	7	AtKMT3;1	SDG8/ASHH2/EFS/CCR1	OsKMT3;1	SDG725	BdKMT3;1	Bradi3g45727	OtKMT3;1	SDG3605

BrKMT3;2	Bra015723	7	AtKMT3;2	SDG26ASHH1	OsKMT3;2	SDG708	BdKMT3;2	Bradi5g10110	OtKMT3;2	SDG3608

BrKMT3;3	Bra010270	8	AtKMT3;3	SDG4/ASHR3	OsKMT3;3a	SDG736	BdKMT3;3a	Bradi3g48497		

BrKMT3;4a	Bra004809	5	AtKMT3;4a	SDG7/ASHH3	OsKMT3;3b	SDG707	BdKMT3;3b	Bradi3g36790		

BrKMT3;4b	Bra00396	7	AtKMT3;4b	SDG24/ASHH4	OsKMT3;4	SDG724	BdKMT3;4a	Bradi1g20527		

BrKMT3;4c	Bra00343	3					BdKMT3;4b	Bradi4g27797		

BrKMT3;4d	Bra007496	9								

BrKMT6A;1	Bra032169	4	AtKMT6A;1	SDG1/CLF	OsKMT6A;1	SDG711	BdKMT6A;1	Bradi1g48340	OtKMT6A	SDG3609

BrKMT6A;2	Bra036300	9	AtKMT6A;2	SDG10/EZA1/SWN	OsKMT6A;2	SDG718	BdKMT6A;2	Bradi1g64460		

BrKMT6A;3a	Bra033334	10	AtKMT6A;3	SDG5/MEA						

BrKMT6A;3b	Bra032592	9								

BrKMT6B;1a	Bra009403	10	AtKMT6B;1	SDG15/ATXR5	OsKMT6B;1	SDG720	BdKMT6B;1	Bradi2g61717		

BrKMT6B;1b	Bra028618	2	AtKMT6B;2	SDG34/ATXR6	OsKMT6B;2	SDG730	BdKMT6B;2	Bradi3g02220		

BrKMT6B;2a	Bra009752	6								

BrKMT6B;2b	Bra029401	2								

BrKMT7;1a	Bra037086	5	AtKMT7;1	SDG2/ATXR3	OsKMT7;1	SDG701	BdKMT7;1	Bradi3g15410	OtKMT7;1a	SDG3606

BrKMT7;1b	Bra039562	1							OtKMT7;1b	SDG3602

BrKMT7;1c	Bra020935	8								

BrS-ET;1	Bra024688	9	AtS-ET;1	SDG35	OsS-ET;1	SDG739	BdS-ET;1	Bradi1g73790	OtS-ET;1a	SDG3615

BrS-ET;2	Bra028726	2	AtS-ET;2	SDG38	OsS-ET;2	SDG741	BdS-ET;3	Bradi5g22730	OtS-ET;1b	SDG3613

BrS-ET;3	Bra031313	5	AtS-ET;3	SDG36	OsS-ET;3	SDG722	BdS-ET;4a	Bradi3g17710	OtS-ET;1c	SDG3611

BrS-ET;4a	Bra036713	9	AtS-ET;4a	SDG39	OsS-ET;4a	SDG740	BdS-ET;4b	Bradi1g11010	OtS-ET;3	SDG3604

BrS-ET;4b	Bra002100	10	AtS-ET;4b	SDG37	OsS-ET;4b	SDG716				

*ud, undetermined

### *B. rapa *SET-domain proteins can be grouped into six classes

**Figure 1:**
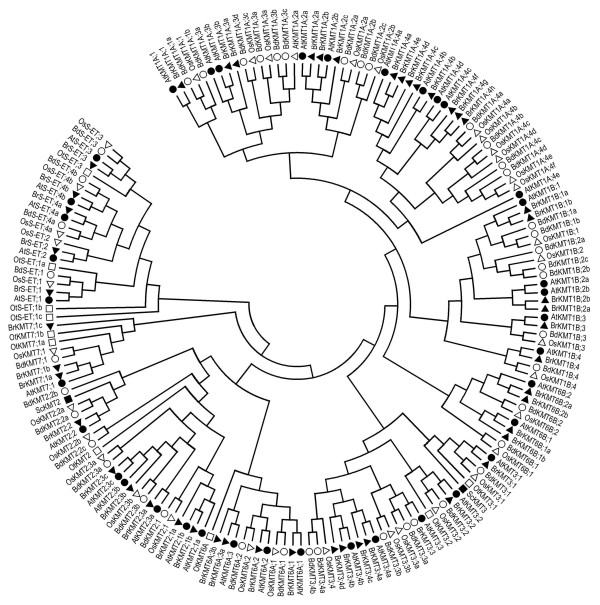
**Phylogenetic tree of SET-domain proteins**. The SET domain sequences of the 175 different proteins were aligned using ClustalW, and the phylogenetic tree analysis was performed using MEGA4. Closed circles and triangles indicate *Arabidopsis thaliana *(At) and *Brassica rapa *(Br) proteins, respectively; open circles and triangles indicate *Brachypodium distachyon *(Bd) and *Oryza sativa *(Os) proteins, respectively; open and closed squares indicate *Ostreococcus tauri *(Ot) and *Saccharomyces cerevisiae *(Sc) proteins, respectively.

To analyze the *B. rapa *SET-domain protein sequences, we extracted SET-domain proteins from several other green lineage species, including 37 proteins from *A. thaliana*, 36 proteins from *O. sativa*, 41 proteins from *B. distachyon*, and 10 proteins from *Ostreococcus tauri *(Table 1). We also included the *Saccharomyces cerevisiae *ScKMT2/Set1 and ScKMT3/Set2 proteins, which are H3K4- and H3K36-specific KMTs, respectively [18,19], and can be used to represent ancient eukaryotic SET-domain proteins from an evolutionary point of view. Phylogenetic analysis of the aforementioned 175 SET-domain proteins revealed that they could be grouped into 6 distinct classes, namely KMT1, KMT2, KMT3, KMT6, KMT7 and S-ET class (Figure 1). The first four class numbers used here are consistent with the nomenclature previously proposed for yeast and animal KMTs [17]. Furthermore, two plant-specific subclasses (namely A and B) were identified for KMT1 and KMT6. Representative members of each class/subclass are found in *A. thaliana*, *B. rapa*, *O. sativa *and *B. distachyon*. The S-ET class members contain an interrupted SET domain and are likely involved in methylation of nonhistone proteins, e.g. RUBISCO subunits; however, their biological functions remain largely unknown. Hereafter, we focused on the KMT classes/subclasses that are involved in histone methylation.

### The KMT1A subclass proteins

**Figure 2:**
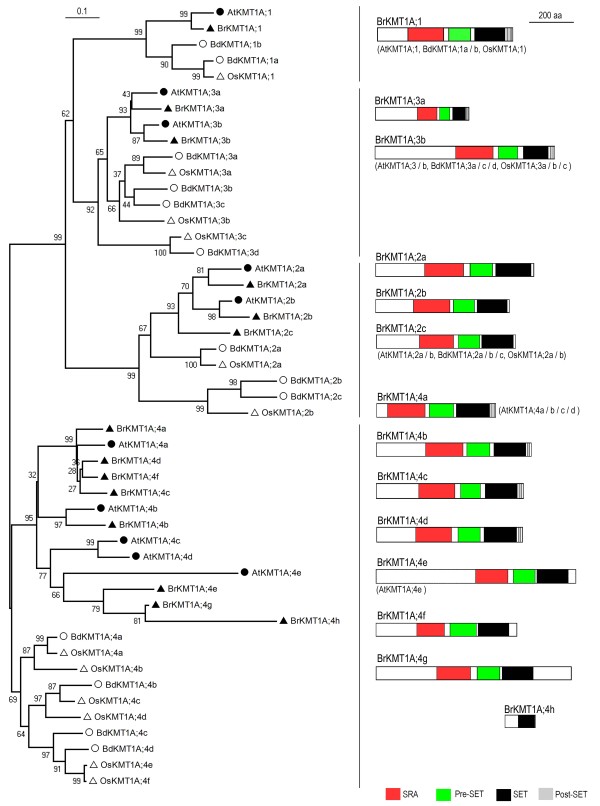
**Domain organization of the KMT1A subclass proteins**. Schematic diagrams show the domain organization of KMT1A proteins and are placed on the right side of the phylogenetic tree. The scale bar indicates the evolutionary distance, the number along the tree branch indicates bootstrap value, and the other information about tree construction and symbol indication can be found in legend of Figure 1. Different conserved protein domains (SRA, Pre-SET, SET, and Post-SET) are colored as indicated. The protein is indicated on top of the schematic diagram and other similar domain organization proteins are indicated in parentheses.

The KMT1A subclass is the largest class and can be further divided into 4distinct groups (Figure 2). In each group, proteins from dicots (*B. rapa *and *A. thaliana*) and monocots (*O. sativa *and *B. distachyon*) clearly fall into separate branches, indicating that they are derived from a common ancestral gene but diverged before the monocot/dicot separation. The first three groups have a relatively simple relationship and small number of genes, but Group-4 is more complex: each plant species has 4-8 members that diverged at various times during evolution. In the case of *B. rapa*, among the 8 members belonging to Group-4, BrKMT1A;4a, BrKMT1A;4c, BrKMT1A;4d, and BrKMT1A;4f are clustered with the *Arabidopsis *AtKMT1A;4a/SDG32/SUVH1; BrKMT1A;4b with AtKMT1A;4b/SDG19/SUVH3; and BrKMT1A;4e, BrKMT1A;4 g and BrKMT1A;4 h with AtKMT1A;4e/SDG11/SUVH10 (Figure 2). Examination of synteny between *B. rapa *and *A. thaliana *(http://brassicadb.org/brad/searchSynteny.php) revealed that *BrKMT1A;4a*, *BrKMT1A;4c *and *BrKMT1A;4d *but not *BrKMT1A;4f *are syntenic with *AtKMT1A;4a/SDG32/SUVH1*, and *BrKMT1A;4 h *but not *BrKMT1A;4e *nor *BrKMT1A;4 g *is syntenic with *AtKMT1A;4e/SDG11/SUVH10*. It thus appears that multiple duplication events occurred, in either a chromosome segment or single gene scale, resulting in more recent amplification of Group-4 genes in *B. rapa *after separation from *A. thaliana *during evolution. In agreement with previous studies in *Arabidopsis*, rice and maize [11,12], few introns are present in *BrKMT1A *genes (Additional File 1: Figure S1). Most *BrKMT1A *genes are represented by ESTs, but some do not have any ESTs in current databases (Additional File 2: Table S1). Our RT-PCR analysis revealed that indeed two genes that lack ESTs, *BrKMT1A;2a *and *BrKMT1A;2c*, are very weakly expressed. Strong expression was detected for *BrKMT1A;4a, *but relatively weak expression was detected for *BrKMY1A;4d *and expression was undetectable for *BrKMT1A;4c *(Additional File 3: Figure S2). Together, these data indicate that expression levels of different *BrKMT1A *genes varied considerably and thus these genes may regulate genome function to different degrees.

The plant KMT1A subclass proteins show high sequence similarity to the animal KMT1 proteins both within the SET domain and in the surrounding regions known as the Pre-SET and post-SET domains. Additionally, most of the plant proteins contain a specific domain named SRA (SET and RING associated). Similar to previously studied *Arabidopsis *proteins [12,20], most of the BrKMT1A proteins also contain SRA, Pre-SET, SET and post-SET domains (Figure 2). These domains are missing in some of the Group-4 proteins; for example, BrKMT1A.4e, BrKMT1A;4f and BrKMT1A;4 g lack a Post-SET domain, and BrKMT1A.4 h lacks SRA, Pre-SET and post-SET domains (Figure 2). Several functions have been reported for SRA domains, including binding with the N-terminal tail of histone H3 and with DNA cytosine methylation [21]. The crystal structure of AtKMT1A;3a/SDG9/SUVH5 revealed that SRA recognizes the methylation status of CG and CHH sequences [22]. The Pre-SET domain contains 9 conserved cysteines. The Post-SET domain is a small cysteine-rich region often found at the C-terminal side of SET domains. Both Pre-SET and Post-SET domains have been shown to affect histone methyltransferase activity of the SET domain [23,24].

Members of the plant KMT1A subclass, like animal KMT1 proteins, are likely to be responsible for H3K9 methylation, an epigenetic mark involved in heterochromatin formation and gene silencing. Consistent with this, analysis of *AtKMT1A;1/SDG33/SUVH4/KYP*, *AtKMT1A;2a/SDG3/SUVH2*, *AtKMT1A;3a/SDG9/SUVH5 *and *AtKMT1A;3b/SDG23/SUVH6 *has revealed their important roles in H3K9 methylation, in heterochromatic gene silencing and in cross-talk between H3K9 and DNA methylation [9,21,22,25-28]. Work in rice also confirmed that several members of this subclass are involved in H3K9 methylation and in transposon silencing [29-31]. Some of the *BrKMT1A *genes might also have similar functions.

### The KMT1B subclass proteins

**Figure 3:**
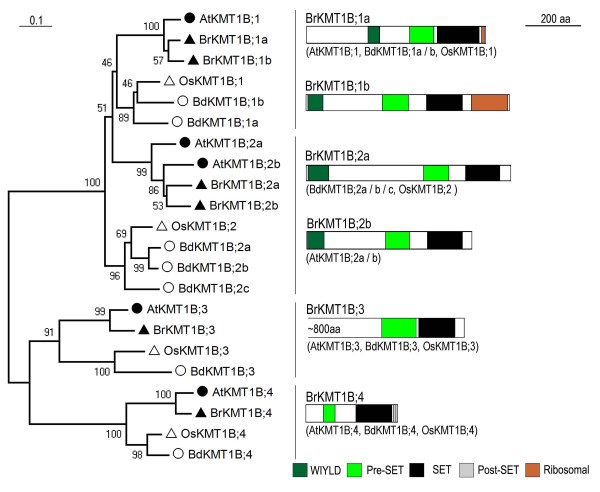
**Domain organization of the KMT1B subclass proteins**. Schematic diagrams show the domain organization of KMT1B proteins and are placed on the right side of the phylogenetic tree. The scale bar indicates the evolutionary distance, the number along the tree branch indicates bootstrap value, and the other information about tree construction and symbol indication can be found in legend of Figure 1. Different conserved protein domains (WIYL, Pre-SET, SET, Post-SET, and Ribosomal) are colored as indicated. The KMT1B;3-group proteins are large in size; therefore the corresponding schematic diagram is drawn with a remove of ~800 aa from the N-terminus, a region without any detectable known protein domains. The protein is indicated on top of the schematic diagram and other similar domain organization proteins are indicated in parentheses.

Six *B. rapa *proteins belong to the KMT1B subclass, which can be further divided into 4 groups (Figure 3). Group-1 contains two *B. rapa *proteins (BrKMT1B;1a and BrKMT1B;1b) whose genes show synteny with *AtKMT1B;1/SDG31/SUVR4*. Moreover, sequence analysis showed that BrKMT1B;1b and AtKMT1B;1/SDG31/SUVR4 have highly similar protein domain organization, indicating that *BrKMT1B;1b *is more conserved and *BrKMT1B;1a *diverged relatively late during evolution after the *B. rapa/A. thaliana *separation. Group-2 has two *B. rapa*, two *A. thaliana *proteins, one *O. sativa *protein, and three *B. distachyon *proteins. Both *BrKMT1B;2a *and *BrKMT1B;2b *show synteny with *AtKMT1B;2b/SDG18/SUVR2*. Group-3 and 4 each have one representative member in each of the four examined higher plant species.

The KMT1B subclass differs from the KMT1A subclass in protein domain organization; specifically, these proteins lack the SRA domain (Figure 3). A recent study demonstrated that AtKMT1B;1/SDG31/SUVR4 possesses H3K9-methyltransferase activities and its binding with ubiquitin converts H3K9me1 to H3K9me3 deposition on transposon chromatin [32]. Notably, the WIYLD domain, which binds ubiquitin, is conserved in BrKMT1B;1a, BrKMT1B;1b, BrKMT1B;2a and BrKMT1B;2b (Figure 3). It was reported that AtKMT1B;3/SDG6/SUVR5/AtCZS is involved in regulation of flowering time, possibly through deposition of H3K9 methylation at the flowering time repressor *FLC *[33]. The functions of other members of the KMT1B subclass remain uncharacterized so far.

### The KMT2 class proteins

**Figure 4:**
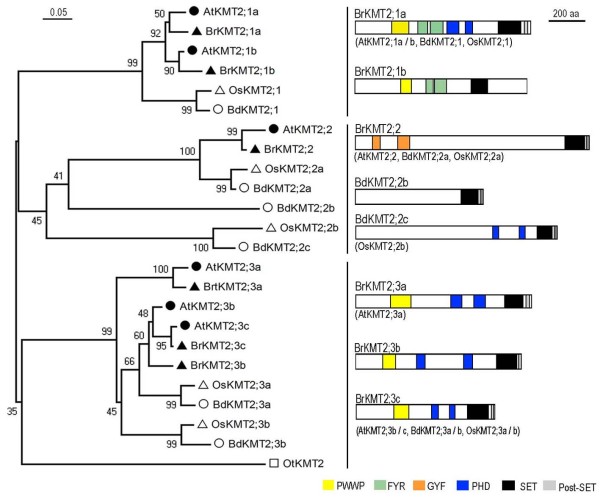
**Domain organization of the KMT2 class proteins**. Schematic diagrams show the domain organization of KMT2 proteins and are placed on the right side of the phylogenetic tree. The scale bar indicates the evolutionary distance, the number along the tree branch indicates bootstrap value, and the other information about tree construction and symbol indication can be found in legend of Figure 1. Different conserved protein domains (PWWP, FYR, GYF, PHD, SET, and Post-SET) are colored as indicated. The protein is indicated on top of the schematic diagram and other similar domain organization proteins are indicated in parentheses.

The KMT2 class includes six *B. rapa *and six *A. thaliana *proteins in 3 groups (Figure 4). This class features highly conserved SET and Post-SET domains with the yeast H3K4-methyltransferase ScKMT2/Set1. Nevertheless, some plant proteins have acquired specific domains during evolution, namely PWWP, PHD, FYR and/or GYF. The PWWP domain is also found in eukaryotic proteins involved in DNA methylation, DNA repair, and regulation of transcription [34], and regulates cell growth and differentiation by mediating protein-protein interactions [35]. The PHD domain is found in a number of chromatin-associated proteins and is thought to be involved in protein-protein interactions important for the assembly of multiprotein complexes [36]. The PWWP domain of the animal BRPF1 protein binds H3K36me3 [35], and the PHD domain is also an important module in proteins that read histone modifications [37]. The FYR domain is composed of FYR-C and FYR-N terminal portions, which are often located close to each other but can also be separated [38]. The GYF domain is proposed to be involved in recognition of proline-rich sequences in protein-protein interactions [39].

KMT2 Group-1 members contain one PWWP, one FYR and two PHD domains. Only one member belonging to Group-1 is found in *O. sativa *or *B. distachyon*, but two members are found in *B. rapa *and *A. thaliana*. Our examination revealed that *BrKMT2;1a *has synteny with *AtKMT2;1a/SDG27/ATX1*, and *BrKMT2;1b *with *AtKMT2;1b/SDG30/ATX2*, suggesting that they are derived from two different ancestral copies before the *B. rapa/A. thaliana *separation. Consistent with this, the *atx1 *mutant plants exhibit strong and pleiotropic defects [40], but the *atx2 *mutant plants have a normal phenotype [41]. The *atx2 *mutation can enhance *atx1 *in reduction of expression of the flowering repressor gene *FLC *through reduced levels of H3K4me3 at the *FLC *locus [42].

The PWWP and FYR domains are absent from the Group-2 members and the PHD domain is found only in some monocot proteins (Figure 4). Only one Group-2 representative member is found in the dicot species *B. rapa *or *A. thaliana*, but the monocot *O. sativa *has two members and *B. distachyon *has three members. The *B. rapa *and *A. thaliana *proteins, as well as one member each from *O. sativa *and *B. distachyon, *contain a GYF domain in the N-terminal part of the protein (Figure 4). The fact that this domain is conserved in KMT2;2 proteins from all four higher plant species suggests that the acquisition of the GYF domain occurred before the monocot/dicot separation and may have a conserved function in higher plants. Genetic analysis demonstrated that *AtKMT2;2*/*SDG25/ATXR7 *is necessary in preventing early flowering [43,44]. The recombinant AtKMT2;2/SDG25/ATXR7 protein was shown to methylate histone H3 *in vitro *and the depletion of AtKMT2;2/SDG25/ATXR7 *in planta *slightly reduced H3K4 and H3K36 methylation at *FLC *chromatin [43,44]. OsKMT2;2b/SDG732 and BdKMT2;2c contain two PHD domains, but BdKMT2;2b, like the yeast protein ScKMT2/Set1, does not contain a recognizable PHD domain. Future study of these monocot proteins will likely provide a deeper understanding of the domain evolution of KMT2 proteins.

The Group-3 KMT2 proteins have a domain organization more similar to Group-1 except that they lack the FYR domain (Figure 4). *B. rapa *and *A. thaliana *both have three Group-3 members, but each of the other two higher plant species has only two Group-3 members. Synteny was observed for *BrKMT2;3c *with *AtKMT2;3c/SDG29/ATX5 *but not with *AtKMT2;3b/SDG16/ATX4*, suggesting that *AtKMT2;3b/SDG16/ATX4 *was derived from a relatively recent duplication event. This is in agreement with a previous study revealing that *AtKMT2;3b/SDG16/ATX4 *and *AtKMT2;3c/SDG29/ATX5 *are collinearly duplicated with *AtKMT2;1a/SDG27/ATX1 *and *AtKMT2;1b/SDG30/ATX2 *[12]. To date, none of the Group-3 proteins has been functionally characterized.

### The KMT3 class proteins

**Figure 5:**
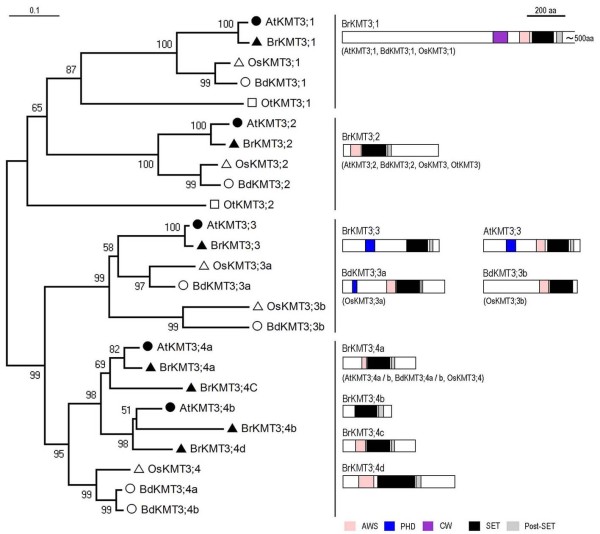
**Domain organization of the KMT3 class proteins**. Schematic diagrams show the domain organization of KMT3 proteins and are placed on the right side of the phylogenetic tree. The scale bar indicates the evolutionary distance, the number along the tree branch indicates bootstrap value, and the other information about tree construction and symbol indication can be found in legend of Figure 1. Different conserved protein domains (AWS, PHD, CW, SET, and Post-SET) are colored as indicated. The KMT3;1-group proteins are large in size; therefore the corresponding schematic diagram is drawn with a remove of ~500 aa from the C-terminus, a region without any detectable known protein domains. The protein is indicated on top of the schematic diagram and other similar domain organization proteins are indicated in parentheses.

The KMT3 class contains 5 members in *A. thaliana *but 7 members in *B. rapa*, and these can be further divided into four groups (Figure 5). The other groups contain a single member per plant species, but Group-4 contains 2 members in *A. thaliana *and 4 members in *B. rapa*. Our examination indicates that *BrKMT3;4a *and *BrKMT3;4c *are syntenic with *AtKMT3;4a*/*SDG7/ASHH3, *and *BrKMT3;4b *and *BrKMT3;4d *with *AtKMT3;4b*/*SDG24/ASHH4*. The ESTs found in the current databases match all four *BrKMT3;4 *genes (Additional File 2: Table S1), and thus do not allow us to distinguish expression of each gene. Our RT-PCR analysis indicated that *BrKMT3;4a *and *BrKMT3;4c *are expressed at higher levels and more broadly in different examined organs/tissues, whereas only weak expression was detected for *BrKMT3;4b *and *BrKMT3;4d *in some organs/tissues (Additional File 3: Figure S2).

The KMT3 class plant proteins share high sequence similarity and share the AWS (a subdomain of Pre-SET, [45]), SET and Post-SET domain organization with the yeast H3K36-methyltransferase ScKMT3/Set2 (Figure 5). The Group-1 proteins have a long sequence and contain an additional CW domain specific to this group. The CW domain of AtKMT3;1/SDG8/ASHH2/EFS/CCR1 was recently shown to bind H3K4me1/me2 [46], suggesting a novel link between H3K4 and H3K36 methylation in plants. AtKMT3;1/SDG8/ASHH2/EFS/CCR1 is the major H3K36-methyltransferase specifically required for H3K36me2 and H3K36me3 deposition, and activates expression of hundreds of genes including *FLC *and *MAF*s [47]. Depletion of AtKMT3;1/SDG8/ASHH2/EFS/CCR1 causes pleiotropic phenotypes, including early flowering, reduced organ size, increased shoot branching, perturbed fertility and carotenoid composition, and impaired plant defenses against pathogens [47-54]. The other group of KMT3 plant proteins have a shorter sequence and do not contain the CW domain; interestingly the depletion of AtKMT3;2/SDG26/ASHH1 resulted in a late-flowering phenotype associated with elevated levels of *FLC *expression [47]. The Group-3 KMT3 proteins, with the exception of BdKMT3;3b and OsKMT3;3b/SDG707, contain a PHD domain; and AtKMT3;3/SDG4/ASHR3 was reported to be involved in pollen and stamen development possibly through mediating H3K4me2 and H3K36me3 deposition [55,56]. The functions of the Group-4 proteins remain unexamined so far. Examination of this group in *B. rapa *could be a challenge because of gene multiplication and more diverged sequences (Figure 5).

### The KMT6A subclass proteins

**Figure 6:**
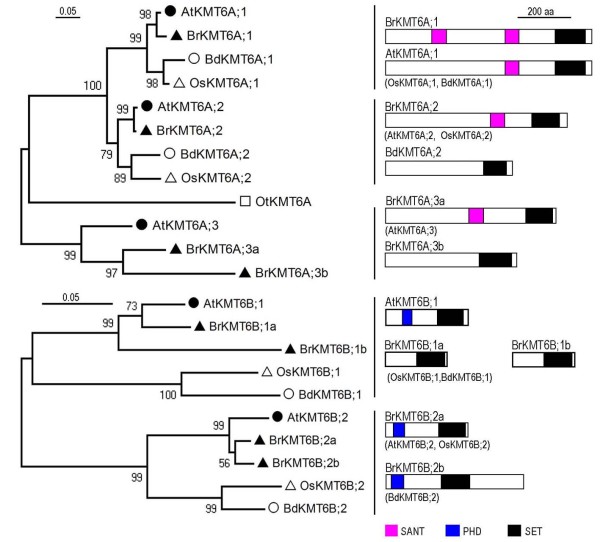
**Domain organization of the KMT6 class proteins**. The KMT6 class can be divided into 2 subclasses (**a **and **b**). Schematic diagrams show the domain organization of KMT6 proteins and are placed on the right side of the phylogenetic tree. The scale bar indicates the evolutionary distance, the number along the tree branch indicates bootstrap value, and the other information about tree construction and symbol indication can be found in legend of Figure 1. Different conserved protein domains (SANT, PHD, and SET) are colored as indicated. The protein is indicated on top of the schematic diagram and other similar domain organization proteins are indicated in parentheses.

The KMT6A subclass includes 4 members in *B. rapa *and 3 well-characterized members in *A. thaliana*, AtKMT6A;1/SDG1/CLF, AtKMT6A;2/SDG10/EZA1/SWN and AtKMT6A;3/SDG5/MEA, which represent three distinct groups (Figure 6a). *AtKMT6A;1/SDG1/CLF *and *AtKMT6A;2/SDG10/EZA1/SWN *are broadly expressed and partially redundant in regulation of vegetative and reproductive development, whereas *AtKMT6A;3/SDG5/MEA *appears to function specifically in gametophyte and seed development [7,8,57]. The three *Arabidopsis *proteins can act as a key component of the evolutionarily conserved Polycomb Repressive Complex 2 (PRC2), which trimethylates H3K27 involved in transcriptional repression [57]. AtKMT6A;1/SDG1/CLF and AtKMT6A;2/SDG10/EZA1/SWN represent Group-1 and Group-2, respectively, and each has an orthologue in different higher plant species. AtKMT6A;3/SDG5/MEA, which belongs to Group-3, has no orthologue in monocots but has two orthologues in *B. rapa*. Both *BrKMT6A;3a *and *BrKMT6A;3b *are syntenic with *AtKMT6A;3/SDG5/MEA*, and BrKMT6A;3a has a more similar protein domain organization to AtKMT6A;3/SDG5/MEA than does BrKMT6A;3b, suggesting that *BrKMT6A;3b *may have diverged after the *A. thaliana*/*B. rapa *separation during evolution.

The SANT (SWI3, ADA2, N-CoR, and TFIIIB DNA-binding) domain is found in most of the plant KMT6A subclass proteins. This domain is also found in a number of other chromatin remodeling proteins with multiple activities such as DNA-binding, histone tail binding, and protein-protein interactions [58]. Nevertheless, the precise role of the SANT domain in KMT6A proteins is currently unknown. Notably, BrKMT6A;3b does not contain a SANT domain. As expected from restricted *AtKMT6A;3/SDG5/MEA *expression in only a small number of cells during reproduction, expression of both *BrKMT6A;3a *and *BrKMT6A;3b *is barely detectable in the examined tissues (Additional File 3: Figure S2). It will be interesting to investigate *BrKMT6A;3a *and *BrKMT6A;3b *expression during reproduction and to examine whether both genes are functionally important.

### The KMT6B subclass proteins

The KMT6B subclass includes two members each in *A. thaliana*, *B. distachyon *and *O. sativa*, and four members in *B. rapa*, which together can be divided into two distinct groups (Figure 6b). The two members from *A. thaliana*, AtKMT6B;1/SDG15/ATXR5 and AtKMT6B;2/SDG34/ATXR6, were classified as trithorax-related in the first genome analysis of *Arabidopsis *SET-domain proteins [11]. However, our study as well as two previous studies that included a more complete set of plant SET-domain proteins clearly show that AtKMT6B;1/SDG15/ATXR5 and AtKMT6B;2/SDG34/ATXR6 belong to the KMT6B subclass (Figure 1) [12,20]. Consistent with this, functional analysis revealed that AtKMT6B;1/SDG15/ATXR5 and AtKMT6B;2/SDG34/ATXR6 are involved in monomethylation of H3K27 [59]. They appear to act redundantly, because depletion of H3K27 monomethylation is only detectable in the *atxr5 atxr6 *double mutant [59]. KMT6A-mediated H3K27me3 is mainly present in euchromatic regions and is important for gene silencing [58], but KMT6B-mediated H3K27me1 is found in heterochromatic chromocenters and is important for heterochromatin condensation and replication in *Arabidopsis *[60].

Distinct from KMT6A proteins containing a SANT domain, many plant KMT6B subclass proteins contain a PHD domain (Figure 6). The PHD domain of both AtKMT6B;1/SDG15/ATXR5 and AtKMT6B;2/SDG34/ATXR6 strongly bind unmethylated H3 tail peptides (amino acids 1-21), and this binding is negatively affected by methylation on H3K4 [60]. This binding preference may help to assure that these KMT6B proteins are not targeted to euchromatin and active genes enriched in H3K4 methylation. Remarkably, both Group-1 and Group-2 members are duplicated in *B. rapa*. Both *BrKMT6B;1a *and *BrKMT6B;1b *are syntenic with *AtKMT6B;1/SDG15/ATXR5, *and both *BrKMT6B;2a *and *BrKMT6B;2b *with *AtKMT6B;2/SDG34/ATXR6*. Expression of *BrKMT6B;1a*, *BrKMT6B;2a *and *BrKMT6B;2b *was detected in different tissues, but we failed to detect *BrKMT6B;1b *expression (Additional File 3: Figure S2). It is reasonable to speculate that *BrKMT6B;1a*, *BrKMT6B;2a *and *BrKMT6B;2b *might have redundant functions.

### The KMT7 class proteins

**Figure 7:**
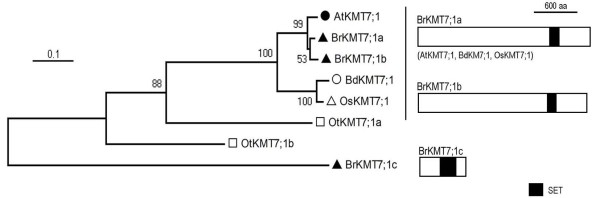
**Domain organization of the KMT7 class proteins**. Schematic diagrams show the domain organization of KMT7 proteins and are placed on the right side of the phylogenetic tree. The scale bar indicates the evolutionary distance, the number along the tree branch indicates bootstrap value, and the other information about tree construction and symbol indication can be found in legend of Figure 1. The conserved SET-domain is indicated. The protein is indicated on top of the schematic diagram and other similar domain organization proteins are indicated in parentheses.

The KMT7 class contains a single member each in *A. thaliana*, *O. sativa *and *B. distachyon*, but three members in *B. rapa *(Figure 7). Although the *Arabidopsis *protein AtKMT7;1/SDG2/ATXR3 was considered to be related to members of the KMT2 class in some previous studies [11,20], it was located outside of any classes in the phylogenetic tree analysis by Springer and colleagues [12], and our analysis here revealed that it is grouped together with some other green lineage proteins, forming the plant KMT7 class (Figure 1). Unlike the classes described above, the plant and animal KMT7 classes do not cluster although they are predicted to have similar functions in H3K4 methylation. Representatives of the animal KMT7 class are only found in mammals and include the human SET7/9, which monomethylates H3K4 and also methylates a number of nonhistone proteins [17]. The plant KMT7 proteins did not show the highest sequence similarities with the human SET7/9, and depletion of AtKMT7;1/SDG2/ATXR3 resulted in a global reduction of H3K4me3 and caused pleiotropic defects in both sporophyte and gametophyte development [61,62]. Both *BrKMT7;1a *and *BrKMT7;1b *but not *BrKMT7;1c *have synteny with *AtKMT7;1/SDG2/ATXR3*, and phylogenetic analysis showed that BrKMT7;1a is more closely related to AtKMT7;1/SDG2/ATXR3. RT-PCR analysis revealed that *BrKMT7;1b *is expressed at a higher level than *BrKMT7;1a *(Additional File 3: Figure S2). In view of the important function of *AtKMT7;1a/SDG2/ATXR3*, it will be interesting to investigate roles of *BrKMT7;1a *and *BrKMT7;1b *in histone methylation and plant development in *B. rapa*.

## Discussion

Over last 10 years, a number of SET-domain genes in *Arabidopsis *and in rice have been characterized and shown to exert crucial chromatin-based functions *via *histone methylation during plant growth and development [3,15]. However, the nomenclature of plant SET-domain proteins remains complex, and multiple synonyms exist for many *Arabidopsis *proteins (Table 1), which could cause considerable confusion in this important field. Nomenclature based on sequence similarity has several advantages, informing the prediction of KMT enzyme substrate specificity for its histone lysine residue and providing a global view of KMT types in an organism once its whole genome sequence becomes available. However, the SDG nomenclature failed to provide information concerning enzyme substrate specificity and the number following "SDG" could be long and difficult to remember [12,63], e.g., the first SDG from *B. rapa *(ID = 197) would have been named SDG19701. While the nomenclature by Baumbusch and colleagues provided information about homology to animal proteins, an incomplete list of *Arabidopsis *SET-domain proteins and the limitation (at that time) of having only one plant species with a genome-wide analysis restricted the precision and correctness of phylogenetic grouping in this study [11]. In addition, animal KMT nomenclature had also been noncoherent; a rational nomenclature was proposed only recently [17]. Therefore, the nomenclature we propose here is in line with the latest advances in the field.

**Figure 8:**
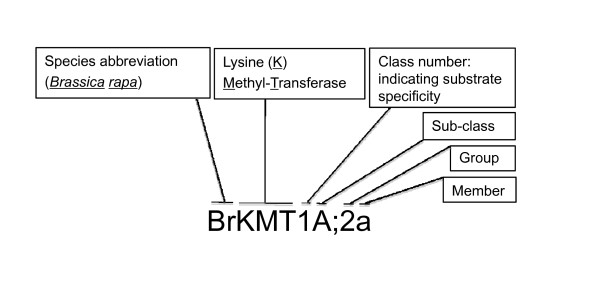
**Nomenclature for plant KMTs**. The BrKMT1A;2a protein serves as an example to show assignment of various layers of information within the nomenclature of a plant KMT. Refer to text for class, subclass, group and member definition.

In accordance with the guidelines of the Commission on Plant Gene Nomenclature [64], the nomenclature of plant KMTs is defined by species initials (e.g. Br for *Brasica rapa*) before KMT, which is followed by the class number (Figure 8). The class number is based on the yeast and animal systems indicating the enzyme substrate specificity, i.e. KMT1 for H3K9, KMT2 for H3K4, KMT3 for H3K36, KMT6 for H3K27, and KMT7 also for H3K4 [17]. Multiple subclasses are indicated by upper-case letters (e.g. KMT1A and KMT1B), and distinct groups within the class/subclass are indicated by an arabic numeral suffix (e.g. KMT1A;1). Members within the group are indicated by lower-case letters (e.g. KMT1A;1a and KMT1A;1b). Subgroups are not currently defined but may be designated in the future as functional analysis and an increasing number of sequenced genomes demonstrate sequence conservation between species or distinct functions for several members of a defined KMT group. The use of a given subgroup suffix should indicate highly similar sequences or equivalent functional roles between several species. The new nomenclature may be difficult to adopt in *Arabidopsis *because the original names for a number of SET-domain proteins are familiar to researchers, but a coherent and rational nomenclature for different species is important and useful because of the enormous interest in KMTs. The guidelines proposed here will be particularly useful for nomenclature of newly identified SET-domain proteins, which are being discovered at an exponentially increasing rate as genome sequences become available for additional plant species.

We identified 49 SET-domain proteins from the recently completed whole genome sequence of *B. rapa*. Among them, 5 proteins belong to the S-ET class likely involved in nonhistone protein methylation, 20 proteins belong to the KMT1 class potentially involved in H3K9 methylation, 6 proteins belong to the KMT2 class potentially involved in H3K4 methylation, 7 proteins belong to the KMT3 class potentially involved in H3K36 methylation, 8 proteins belong to the KMT6 class potentially involved in H3K27 methylation, and 3 belong to the KMT7 class also potentially involved in H3K4 methylation. This *in silico *survey is useful for future functional analysis of this important family of epigenetic regulators in *Brassica*. H4K20 methylation was detected in *Arabidopsis *using antibodies [28,65], but the catalyzing enzyme(s) involved is(are) not yet known and the current phylogenetic analysis did not allow prediction of a specific KMT class involved in H4K20 methylation. It is possible that some members of the aforementioned KMT classes catalyze H4K20 methylation. The total number of KMTs in *B. rapa *(49) is slightly higher than that identified in *A. thaliana *(37), *O. sativa *(36) and B. *distachyon *(41). Nevertheless, we could not exclude the possibility that a few more KMTs may be missing from the currently available genome sequence of *B. rapa*.

Gene duplication is one of the primary driving forces in the evolution of genomes and genetic systems, and is considered to be a major mechanism for the establishment of new gene functions and the generation of evolutionary novelty [66,67]. Contrary to what would be expected from the chromosome number duplication in *B. rapa *compared to *A. thaliana*, the number of KMT genes in *B. rapa *(49) is much less than double the number of *A. thaliana *KMTs (74). Many duplicated genes show synteny with their *A. thaliana *homologues, suggesting that they are derived from chromosome/genome segment duplications. Three alternative outcomes can occur in the evolution of duplicated genes: (i) one copy may simply become silenced by degenerative mutations (nonfunctionalization); (ii) one copy may acquire a novel, beneficial function and become preserved by natural selection (neofunctionalization); (iii) both copies may become partially compromised by mutation so that their total capacity adds up to the capacity of the single-copy ancestral gene (subfunctionalization) [66]. These different outcomes likely apply to different duplicated KMT genes, judging from their expression patterns (Additional File 3: Figure S2). Expression of *BrKMT1A;4c *and *BrKMT6B;1b *was undetectable, suggesting that they might have been nonfunctionalized. The duplicated pairs *BrKMT1B;2a *and *BrKMT1B;2b*, *BrKMT3;4b *and BrKMT3;4d, or *BrKMT7;1a *and *BrKMT7;1b *are differentially expressed in plant organs, suggesting that they might have acquired distinct tissue-specific functions. Finally, expression of some duplicated genes, e.g. *BrKMT1A;2a *and *BrKMT1A;2c*, *BrKMT1B;1a *and *BrKMT1B;1b*, or *BrKMT6A;3a *and *BrKMT6A;3b *showed similar patterns, suggesting that they might be subfunctionalized and/or have redundant functions.

Among the groups showing gene duplications in *B. rapa*, it is worth to note that in *Arabidopsis*, *AtKMT6A;3/SDG5/MEA *is critical for parental gene imprinting and seed development [8,68], *AtKMT6B;1/SDG15/ATX5 *and *AtKMT6B;2/SDG34/ATX6 *are important for heterochromatin condensation and replication in *Arabidopsis *[59,60], and *AtKMT7;1/SDG2/ATXR3 *is essential for both sporophyte and gametophyte development [61,62]. It will be of great interest to investigate these groups of genes for their regulation and function in chromatin organization, plant growth and development in *B. rapa*.

## Conclusions

Our study shows that the plant SET-domain KMT proteins can be phylogenetically grouped into distinct classes and that the classes involved in histone methylation can be named in accordance with the nomenclature proposed for animal and yeast SET-domain KMTs. Such a coherent and rational nomenclature in different organisms will help avoid confusion caused by the existence of multiple names for the same protein or gene. The information provided on the *B. rapa *KMTs will also be beneficial for future research to unravel the mechanisms of epigenetic regulation in *Brassica *crops.

## Methods

### SET-domain protein identification

Sequences of SET-domain proteins from *A. thaliana*, *O. sativa*, *O. tauri *and *S. cerevisiae *were retrieved from the Chromatin Database with the key word SDG in species database respectively (ChromDB, http://www.chromdb.org). These sequences, primarily those from *A. thaliana *and *O. sativa*, were used as queries to search the *B. distachyon *genome (http://www.brachypodium.org) and the *B. rapa *genome (http://brassicadb.org/brad/index.php) by using the BLASTp and tBLASTn tools (http://blast.ncbi.nlm.nih.gov). The Expect threshold was set at 1.0 and other parameters were set at default values. We did not use a strict E-value threshold; rather we examined each of the resulting hits for the presence of the SET or S-ET domain to collect previously unidentified sequences. The synteny analysis was performed using the online viewer tool (http://brassicadb.org/brad/index.php). ESTs of the *B. rapa *SET-domain protein genes were retrieved from the Brassica Database (http://brassicadb.org/brad/index.php) and from NCBI (http://blast.ncbi.nlm.nih.gov), using an Expect threshold of 1, and a minimum sequence length of 50 bp.

### Protein domain organization analysis

The protein sequences were analyzed for domain organization using NCBI-CD searches (http://ncbi.nlm.nih.gov/Structure/cdd/wrpsb.cgi). The low-complexity filter was turned off, and the Expect value was set at 1.0 to detect short domains or regions of less conservation in this analysis. Domains were also verified and named according to the SMART database (http://smart.embl-heidelberg.de/).

### Phylogenetic analysis

Multiple sequence alignments of SET-domain sequences were performed using the ClustalW program [69]. The resulting file was subjected to phylogenic analysis using the MEGA4.0 program [70]. The trees were constructed with the following settings: Tree Inference as Neighbor-Joining; Include Sites as pairwise deletion option for total sequences analysis and complete deletion option for each class analysis; Substitution Model: Poisson correction; and Bootstrap test of 1,000 replicates for internal branch reliability.

### RT-PCR Analysis

*B. rapa *plants were grown at 18-22°C under a 12 h light (10,000 Lx)/12 h dark photoperiod. Leaves were collected from 2-, 4-, 6-, 8- or 10-week-old plants; roots and stems were collected from 6-week-old plants; flower buds were collected from 10-week-old plants. Total RNA was extracted using Trizol reagent (Invitrogen, USA) from about 100 mg of collected plant tissue. The RNA preparation was then treated with DNaseI (Promega, USA) for 30 min at 37°C, followed by enzyme inactivation by incubation at 65°C for 5 min. First strand cDNA was made using an RT-PCR Kit (RevertAid™ First Strand cDNA Synthesis Kit, Fermentas, CA). The RT-solution with first strand cDNA was stored at -80°C. Primers used for the RT-PCR reactions are listed in Additional File 4: Table S2. Conditions for the PCR reactions were as follows: 94°C for 3 min; then 30 cycles of 94°C for 30 s, 50-63°C for 30 s, and 72°C for 1 min; and finally 72°C for 8 min. PCR products were separated in a 1.5% (w/v) agarose Tris-borate/EDTA buffer gel and visualized by ethidium bromide staining.

## Authors' contributions

YH conducted most of the experiments and drafted the manuscript; CL contributed to the RT-PCR experiment and participated in the drafting of the manuscript; WHS and YR conceived and directed the study, and wrote the final version of the manuscript. All authors read and approved the final manuscript.

## Supplementary Material

Additional file 1 **Figure S1**. ORF organization of the *B. rapa *KMT1A-group genes. Click here for file

Additional file 2 **Table S1**. List of ESTs of the *B. rapa *SET-domain genes. Click here for file

Additional file 3 **Figure S2**. Expression analysis of *BrKMT *duplication genes in different organs and stages. Click here for file

Additional file 4 **Table S2**. List of gene specific primers used in this study. Click here for file

## References

[B1] Strahl BD Allis CD The language of covalent histone modifications Nature 2000 403 41 45 10.1038/47412 10638745

[B2] Martin C Zhang Y The diverse functions of histone lysine methylation Nat Rev Mol Cell Biol 2005 6 838 849 10.1038/nrm1761 16261189

[B3] Liu C Lu F Cui X Cao X Histone methylation in higher plants Annu Rev Plant Biol 2010 61 395 420 10.1146/annurev.arplant.043008.091939 20192747

[B4] Tschiersch B Hofmann A Krauss V Dorn R Korge G Reuter G The protein encoded by the *Drosophila *position-effect variegation suppressor gene *Su(var)3-9 *combines domains of antagonistic regulators of homeotic gene complexes EMBO J 1994 13 3822 3831 10.1002/j.1460-2075.1994.tb06693.x PMC395295 7915232

[B5] Qian C Zhou MM SET domain protein lysine methyltransferases: structure, specificity and catalysis Cell Mol Life Sci 2006 63 2755 2763 10.1007/s00018-006-6274-5 PMC11136235 17013555

[B6] Alvarez-Venegas R Sadder M Tikhonov A Avramova Z Origin of the bacterial SET domain genes: vertical or horizontal? Mol Biol Evol 2007 24 482 497 10.1093/molbev/msl184 17148507

[B7] Goodrich J Puangsomlee P Martin M Long D Meyerowitz EM Coupland G A Polycomb-group gene regulates homeotic gene expression in *Arabidopsis* Nature 1997 386 44 51 10.1038/386044a0 9052779

[B8] Grossniklaus U Vielle-Calzada JP Hoeppner MA Gagliano WB Maternal control of embryogenesis by *MEDEA*, a polycomb group gene in *Arabidopsis* Science 1998 280 446 450 10.1126/science.280.5362.446 9545225

[B9] Jackson JP Lindroth AM Cao X Jacobsen SE Control of CpNpG DNA methylation by the KRYPTONITE histone H3 methyltransferase Nature 2002 416 556 560 10.1038/nature731 11898023

[B10] Shen WH NtSET1, a member of a newly identified subgroup of plant SET-domain-containing proteins, is chromatin-associated and its ectopic overexpression inhibits tobacco plant growth Plant J 2001 28 371 383 10.1046/j.1365-313x.2001.01135.x 11737775

[B11] Baumbusch LO Thorstensen T Krauss V Fischer A Naumann K Assalkhou R Schulz I Reuter G Aalen RB The *Arabidopsis thaliana *genome contains at least 29 active genes encoding SET domain proteins that can be assigned to four evolutionarily conserved classes Nucleic Acids Res 2001 29 4319 4333 10.1093/nar/29.21.4319 PMC60187 11691919

[B12] Springer NM Napoli CA Selinger DA Pandey R Cone KC Chandler VL Kaeppler HF Kaeppler SM Comparative analysis of SET domain proteins in maize and *Arabidopsis *reveals multiple duplications preceding the divergence of monocots and dicots Plant Physiol 2003 132 907 925 10.1104/pp.102.013722 PMC167030 12805620

[B13] Pien S Grossniklaus U Polycomb group and trithorax group proteins in *Arabidopsis* Biochim Biophys Acta 2007 1769 375 382 10.1016/j.bbaexp.2007.01.010 17363079

[B14] Shen WH Xu L Chromatin remodeling in stem cell maintenance in *Arabidopsis thaliana* Mol Plant 2009 2 600 609 10.1093/mp/ssp022 19825642

[B15] Berr A Shafiq S Shen WH Histone modifications in transcriptional activation during plant development Biochim Biophys Acta 2011 1809 567 576 10.1016/j.bbagrm.2011.07.001 21777708

[B16] Wang X Wang H Wang J Sun R Wu J Liu S Bai Y Mun JH Bancroft I Cheng F Huang S Li X Hua W Wang J Wang X Freeling M Pires JC Paterson AH Chalhoub B Wang B Hayward A Sharpe AG Park BS Weisshaar B Liu B Li B Liu B Tong C Song C Duran C Peng C Geng C Koh C Lin C Edwards D Mu D Shen D Soumpourou E Li F Fraser F Conant G Lassalle G King GJ Bonnema G Tang H Wang H Belcram H Zhou H Hirakawa H Abe H Guo H Wang H Jin H Parkin IA Batley J Kim JS Just J Li J Xu J Deng J Kim JA Li J Yu J Meng J Wang J Min J Poulain J Wang J Hatakeyama K Wu K Wang L Fang L Trick M Links MG Zhao M Jin M Ramchiary N Drou N Berkman PJ Cai Q Huang Q Li R Tabata S Cheng S Zhang S Zhang S Huang S Sato S Sun S Kwon SJ Choi SR Lee TH Fan W Zhao X Tan X Xu X Wang Y Qiu Y Yin Y Li Y Du Y Liao Y Lim Y Narusaka Y Wang Y Wang Z Li Z Wang Z Xiong Z Zhang Z The genome of the mesopolyploid crop species *Brassica rapa* Nat Genet 2011 43 1035 1039 10.1038/ng.919 21873998

[B17] Allis CD Berger SL Cote J Dent S Jenuwien T Kouzarides T Pillus L Reinberg D Shi Y Shiekhattar R Shilatifard A Workman J Zhang Y New nomenclature for chromatin-modifying enzymes Cell 2007 131 4 633 636 10.1016/j.cell.2007.10.039 18022353

[B18] Briggs SD Bryk M Strahl BD Cheung WL Davie JK Dent SY Winston F Allis CD Histone H3 lysine 4 methylation is mediated by set1 and required for cell growth and rDNA silencing in *Saccharomyces cerevisiae* Genes Dev 2001 15 3286 3295 10.1101/gad.940201 PMC312847 11751634

[B19] Strahl BD Grant PA Briggs SD Sun ZW Bone JR Caldwell JA Mollah S Cook RG Shabanowitz J Hunt DF Allis CD Set2 is a nucleosomal histone H3-selective methyltransferase that mediates transcriptional repression Mol Cell Biol 2002 22 1298 1306 10.1128/mcb.22.5.1298-1306.2002 PMC134702 11839797

[B20] Ng DW Wang T Chandrasekharan MB Aramayo R Kertbundit S Hall TC Plant SET domain-containing proteins: structure, function and regulation Biochim Biophys Acta 2007 1769 316 329 10.1016/j.bbaexp.2007.04.003 PMC2794661 17512990

[B21] Johnson LM Bostick M Zhang X Kraft E Henderson I Callis J Jacobsen SE The SRA methyl-cytosine-binding domain links DNA and histone methylation Curr Biol 2007 17 379 384 10.1016/j.cub.2007.01.009 PMC1850948 17239600

[B22] Rajakumara E Law JA Simanshu DK Voigt P Johnson LM Reinberg D Patel DJ Jacobsen SE A dual flip-out mechanism for 5mC recognition by the *Arabidopsis *SUVH5 SRA domain and its impact on DNA methylation and H3K9 dimethylation *in vivo* Genes Dev 2011 25 137 152 10.1101/gad.1980311 PMC3022260 21245167

[B23] Rea S Eisenhaber F O'Carroll D Strahl BD Sun ZW Schmid M Opravil S Mechtler K Ponting CP Allis CD Jenuwein T Regulation of chromatin structure by site-specific histone H3 methyltransferases Nature 2000 406 593 599 10.1038/35020506 10949293

[B24] Tachibana M Sugimoto K Fukushima T Shinkai Y Set domain-containing protein, G9a, is a novel lysine-preferring mammalian histone methyltransferase with hyperactivity and specific selectivity to lysines 9 and 27 of histone H3 J Biol Chem 2001 276 25309 25317 10.1074/jbc.M101914200 11316813

[B25] Malagnac F Bartee L Bender J An *Arabidopsis *SET domain protein required for maintenance but not establishment of DNA methylation EMBO J 2002 21 6842 6852 10.1093/emboj/cdf687 PMC139107 12486005

[B26] Ebbs ML Bartee L Bender J H3 lysine 9 methylation is maintained on a transcribed inverted repeat by combined action of SUVH6 and SUVH4 methyltransferases Mol Cell Biol 2005 25 10507 10515 10.1128/MCB.25.23.10507-10515.2005 PMC1291251 16287862

[B27] Ebbs ML Bender J Locus-specific control of DNA methylation by the *Arabidopsis *SUVH5 histone methyltransferase Plant Cell 2006 18 1166 1176 10.1105/tpc.106.041400 PMC1456864 16582009

[B28] Naumann K Fischer A Hofmann I Krauss V Phalke S Irmler K Hause G Aurich AC Dorn R Jenuwein T Reuter G Pivotal role of *AtSUVH2 *in heterochromatic histone methylation and gene silencing in *Arabidopsis* EMBO J 2005 24 1418 1429 10.1038/sj.emboj.7600604 PMC1142535 15775980

[B29] Ding Y Wang X Su L Zhai J Cao S Zhang D Liu C Bi Y Qian Q Cheng Z Chu C Cao X SDG714, a histone H3K9 methyltransferase, is involved in *Tos17 *DNA methylation and transposition in rice Plant Cell 2007 19 9 22 10.1105/tpc.106.048124 PMC1820975 17259261

[B30] Ding B Zhu Y Bu ZY Shen WH Yu Y Dong AW *SDG714 *regulates specific gene expression and consequently affects plant growth via H3K9 dimethylation J Integr Plant Biol 2010 52 420 430 10.1111/j.1744-7909.2010.00927.x 20377704

[B31] Qin FJ Sun QW Huang LM Chen XS Zhou DX Rice SUVH histone methyltransferase genes display specific functions in chromatin modification and retrotransposon repression Mol Plant 2010 3 773 782 10.1093/mp/ssq030 20566579

[B32] Veiseth SV Rahman MA Yap KL Fischer A Egge-Jacobsen W Reuter G Zhou MM Aalen RB Thorstensen T The SUVR4 histone lysine methyltransferase binds ubiquitin and converts H3K9me1 to H3K9me3 on transposon chromatin in Arabidopsis PLoS Genet 2011 7 e1001325 10.1371/journal.pgen.1001325 PMC3053343 21423664

[B33] Krichevsky A Gutgarts H Kozlovsky SV Tzfira T Sutton A Sternglanz R Mandel G Citovsky V C2H2 zinc finger-SET histone methyltransferase is a plant-specific chromatin modifier Dev Biol 2007 303 259 269 10.1016/j.ydbio.2006.11.012 PMC1831845 17224141

[B34] Qiu C Sawada K Zhang X Cheng X The PWWP domain of mammalian DNA methyltransferase Dnmt3b defines a new family of DNA-binding folds Nat Struct Biol 2002 9 217 224 10.1038/nsb759 PMC4035047 11836534

[B35] Vezzoli A Bonadies N Allen MD Freund SM Santiveri CM Kvinlaug BT Huntly BJ Gottgens B Bycroft M Molecular basis of histone H3K36me3 recognition by the PWWP domain of Brpf1 Nat Struct Mol Biol 2010 17 617 619 10.1038/nsmb.1797 20400950

[B36] Aasland R Gibson TJ Stewart AF The PHD finger: implications for chromatin-mediated transcriptional regulation Trends Biochem Sci 1995 20 56 59 10.1016/s0968-0004(00)88957-4 7701562

[B37] Yun M Wu J Workman JL Li B Readers of histone modifications Cell Res 2011 21 564 578 10.1038/cr.2011.42 PMC3131977 21423274

[B38] Schultz J Copley RR Doerks T Ponting CP Bork P SMART: a web-based tool for the study of genetically mobile domains Nucleic Acids Res 2000 28 231 234 10.1093/nar/28.1.231 PMC102444 10592234

[B39] Kofler MM Freund C The GYF domain FEBS J 2006 273 245 256 10.1111/j.1742-4658.2005.05078.x 16403013

[B40] Alvarez-Venegas R Pien S Sadder M Witmer X Grossniklaus U Avramova Z *ATX-1*, an *Arabidopsis *homolog of *trithorax*, activates flower homeotic genes Curr Biol 2003 13 627 637 10.1016/s0960-9822(03)00243-4 12699618

[B41] Saleh A Alvarez-Venegas R Yilmaz M Le O Hou G Sadder M Al-Abdallat A Xia Y Lu G Ladunga I Avramova Z The highly similar *Arabidopsis *homologs of trithorax *ATX1 *and *ATX2 *encode proteins with divergent biochemical functions Plant Cell 2008 20 568 579 10.1105/tpc.107.056614 PMC2329920 18375658

[B42] Pien S Fleury D Mylne JS Crevillen P Inze D Avramova Z Dean C Grossniklaus U *ARABIDOPSIS TRITHORAX1 *dynamically regulates *FLOWERING LOCUS C *activation via histone 3 lysine 4 trimethylation Plant Cell 2008 20 580 588 10.1105/tpc.108.058172 PMC2329943 18375656

[B43] Berr A Xu L Gao J Cognat V Steinmetz A Dong A Shen WH *SET DOMAIN GROUP25 *encodes a histone methyltransferase and is involved in *FLOWERING LOCUS C *activation and repression of flowering Plant Physiol 2009 151 1476 1485 10.1104/pp.109.143941 PMC2773071 19726574

[B44] Tamada Y Yun JY Woo SC Amasino RM *ARABIDOPSIS TRITHORAX-RELATED7 *is required for methylation of lysine 4 of histone H3 and for transcriptional activation of *FLOWERING LOCUS C* Plant Cell 2009 21 3257 3269 10.1105/tpc.109.070060 PMC2782277 19855050

[B45] Doerks T Copley RR Schultz J Ponting CP Bork P Systematic identification of novel protein domain families associated with nuclear functions Genome Res 2002 12 47 56 10.1101/gr.203201 PMC155265 11779830

[B46] Hoppmann V Thorstensen T Kristiansen PE Veiseth SV Rahman MA Finne K Aalen RB Aasland R The CW domain, a new histone recognition module in chromatin proteins EMBO J 2011 30 1939 1952 10.1038/emboj.2011.108 PMC3098480 21522130

[B47] Xu L Zhao Z Dong A Soubigou-Taconnat L Renou JP Steinmetz A Shen WH Di- and tri- but not monomethylation on histone H3 lysine 36 marks active transcription of genes involved in flowering time regulation and other processes in *Arabidopsis thaliana* Mol Cell Biol 2008 28 1348 1360 10.1128/MCB.01607-07 PMC2258740 18070919

[B48] Kim SY He Y Jacob Y Noh YS Michaels S Amasino R Establishment of the vernalization-responsive, winter-annual habit in *Arabidopsis *requires a putative histone H3 methyl transferase Plant Cell 2005 17 3301 3310 10.1105/tpc.105.034645 PMC1315370 16258034

[B49] Zhao Z Yu Y Meyer D Wu C Shen WH Prevention of early flowering by expression of *FLOWERING LOCUS C *requires methylation of histone H3 K36 Nat Cell Biol 2005 7 1256 1260 10.1038/ncb1329 16299497

[B50] Dong G Ma DP Li J The histone methyltransferase SDG8 regulates shoot branching in *Arabidopsis* Biochem Biophys Res Commun 2008 373 659 664 10.1016/j.bbrc.2008.06.096 18602372

[B51] Cazzonelli CI Cuttriss AJ Cossetto SB Pye W Crisp P Whelan J Finnegan EJ Turnbull C Pogson BJ Regulation of carotenoid composition and shoot branching in *Arabidopsis *by a chromatin modifying histone methyltransferase, SDG8 Plant Cell 2009 21 39 53 10.1105/tpc.108.063131 PMC2648095 19174535

[B52] Grini PE Thorstensen T Alm V Vizcay-Barrena G Windju SS Jorstad TS Wilson ZA Aalen RB The ASH1 HOMOLOG 2 (ASHH2) histone H3 methyltransferase is required for ovule and anther development in *Arabidopsis* PLoS One 2009 4 e7817 10.1371/journal.pone.0007817 PMC2772814 19915673

[B53] Berr A McCallum EJ Alioua A Heintz D Heitz T Shen WH *Arabidopsis *histone methyltransferase SET DOMAIN GROUP8 mediates induction of the jasmonate/ethylene pathway genes in plant defense response to necrotrophic fungi Plant Physiol 2010 154 1403 1414 10.1104/pp.110.161497 PMC2971616 20810545

[B54] Palma K Thorgrimsen S Malinovsky FG Fiil BK Nielsen HB Brodersen P Hofius D Petersen M Mundy J Autoimmunity in *Arabidopsis acd11 *is mediated by epigenetic regulation of an immune receptor PLoS Pathog 2010 6 10 e1001137 10.1371/journal.ppat.1001137 PMC2951382 20949080

[B55] Cartagena JA Matsunaga S Seki M Kurihara D Yokoyama M Shinozaki K Fujimoto S Azumi Y Uchiyama S Fukui K The *Arabidopsis SDG4 *contributes to the regulation of pollen tube growth by methylation of histone H3 lysines 4 and 36 in mature pollen Dev Biol 2008 315 355 368 10.1016/j.ydbio.2007.12.016 18252252

[B56] Thorstensen T Grini PE Mercy IS Alm V Erdal S Aasland R Aalen RB The *Arabidopsis *SET-domain protein ASHR3 is involved in stamen development and interacts with the bHLH transcription factor ABORTED MICROSPORES (AMS) Plant Mol Biol 2008 66 47 59 10.1007/s11103-007-9251-y 17978851

[B57] Zheng B Chen X Dynamics of histone H3 lysine 27 trimethylation in plant development Curr Opin Plant Biol 2011 14 123 129 10.1016/j.pbi.2011.01.001 PMC3081887 21330185

[B58] Zhang D Martyniuk CJ Trudeau VL SANTA domain: a novel conserved protein module in Eukaryota with potential involvement in chromatin regulation Bioinformatics 2006 22 2459 2462 10.1093/bioinformatics/btl414 16877755

[B59] Jacob Y Feng S LeBlanc CA Bernatavichute YV Stroud H Cokus S Johnson LM Pellegrini M Jacobsen SE Michaels SD ATXR5 and ATXR6 are H3K27 monomethyltransferases required for chromatin structure and gene silencing Nat Struct Mol Biol 2009 16 763 768 10.1038/nsmb.1611 PMC2754316 19503079

[B60] Jacob Y Stroud H Leblanc C Feng S Zhuo L Caro E Hassel C Gutierrez C Michaels SD Jacobsen SE Regulation of heterochromatic DNA replication by histone H3 lysine 27 methyltransferases Nature 2010 466 987 991 10.1038/nature09290 PMC2964344 20631708

[B61] Berr A McCallum EJ Menard R Meyer D Fuchs J Dong A Shen WH *Arabidopsis SET DOMAIN GROUP2 *is required for H3K4 trimethylation and is crucial for both sporophyte and gametophyte development Plant Cell 2010 22 3232 3248 10.1105/tpc.110.079962 PMC2990135 21037105

[B62] Guo L Yu Y Law JA Zhang X SET DOMAIN GROUP2 is the major histone H3 lysine 4 trimethyltransferase in *Arabidopsis* Proc Natl Acad Sci USA 2010 107 18557 18562 10.1073/pnas.1010478107 PMC2972934 20937886

[B63] Aquea F Vega A Timmermann T Poupin MJ Arce-Johnson P Genome-wide analysis of the SET DOMAIN GROUP family in Grapevine Plant Cell Rep 2011 30 1087 1097 10.1007/s00299-011-1015-0 21293861

[B64] Price CA Commission on plant gene nomenclature Plant Mol Biol Rep 1993 11 273 274

[B65] Fischer A Hofmann I Naumann K Reuter G Heterochromatin proteins and the control of heterochromatic gene silencing in *Arabidopsis* J Plant Physiol 2006 163 358 368 10.1016/j.jplph.2005.10.015 16384625

[B66] Lynch M Force A The probability of duplicate gene preservation by subfunctionalization Genetics 2000 154 459 473 10.1093/genetics/154.1.459 PMC1460895 10629003

[B67] Cannon SB Mitra A Baumgarten A Young ND May G The roles of segmental and tandem gene duplication in the evolution of large gene families in *Arabidopsis thaliana* BMC Plant Biol 2004 4 10 10.1186/1471-2229-4-10 PMC446195 15171794

[B68] Luo M Bilodeau P Koltunow A Dennis ES Peacock WJ Chaudhury AM Genes controlling fertilization-independent seed development in *Arabidopsis thaliana* Proc Natl Acad Sci USA 1999 96 296 301 10.1073/pnas.96.1.296 PMC15133 9874812

[B69] Thompson JD Higgins DG Gibson TJ CLUSTAL W: improving the sensitivity of progressive multiple sequence alignment through sequence weighting, position-specific gap penalties and weight matrix choice Nucleic Acids Res 1994 22 4673 4680 10.1093/nar/22.22.4673 PMC308517 7984417

[B70] Tamura K Dudley J Nei M Kumar S MEGA4: Molecular Evolutionary Genetics Analysis (MEGA) software version 4.0 Mol Biol Evol 2007 24 1596 1599 10.1093/molbev/msm092 17488738

