# Supplementary figures and images for: Phylogenetic analysis and classification of the Brassica rapa SET-domain protein family
Source: BMC Plant Biol. 2011 Dec 14;11:175. doi: 10.1186/1471-2229-11-175 (PMC3264562; doi:10.1186/1471-2229-11-175)

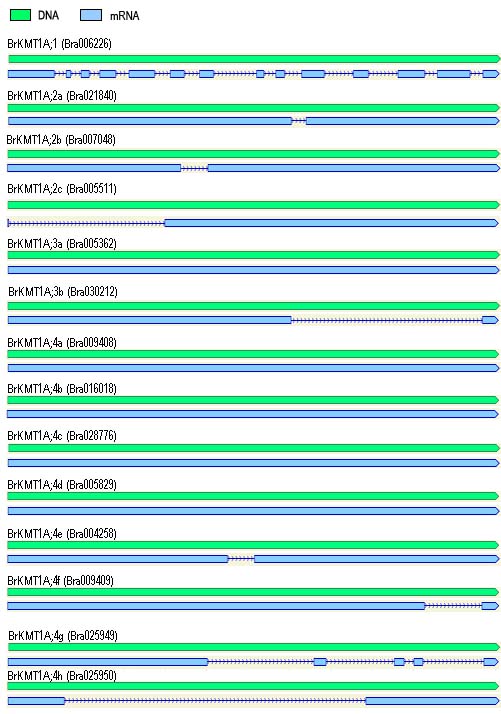

Supplement: Additional file 1 — Figure S1. ORF organization of the B. rapa KMT1A-group genes. [file 1471-2229-11-175-S1.JPEG]

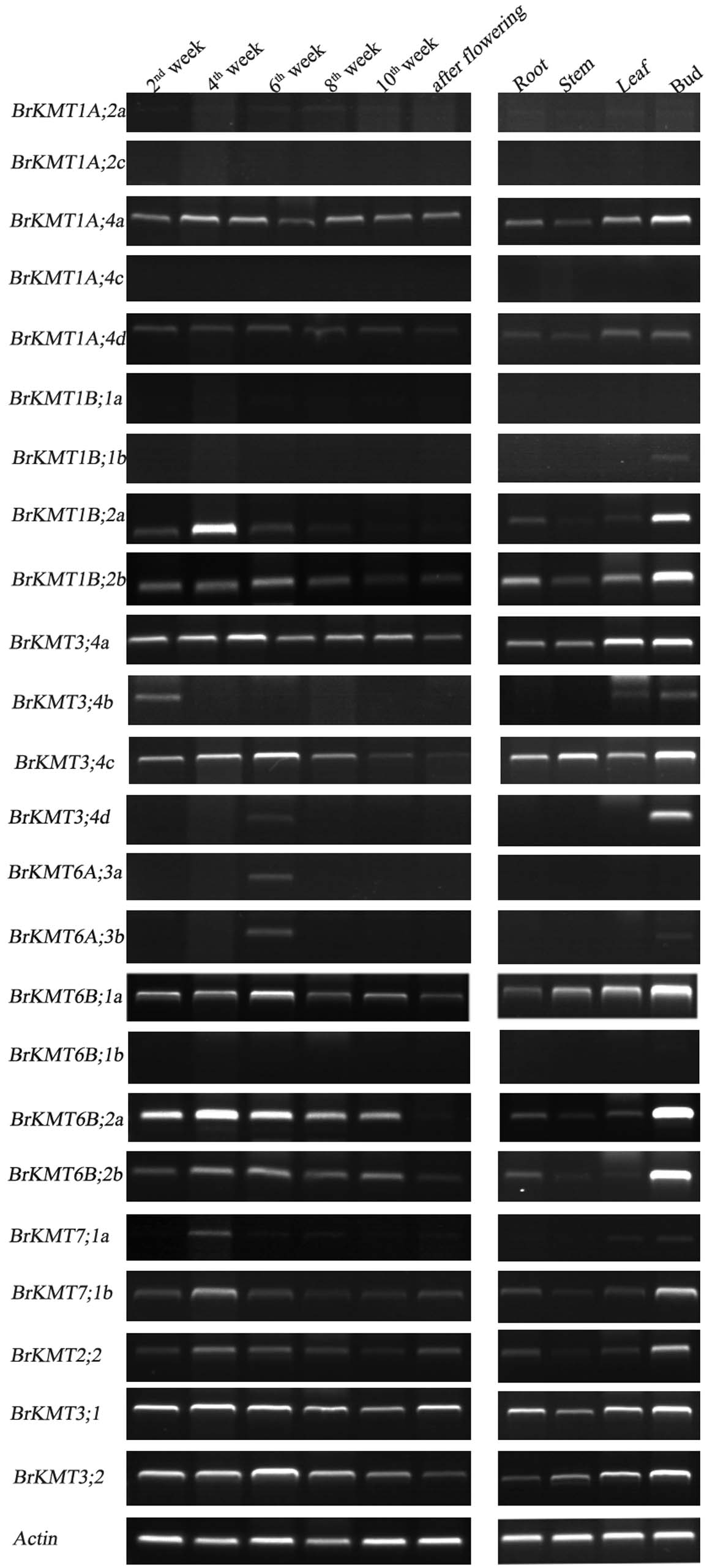

Supplement: Additional file 3 — Figure S2. Expression analysis of BrKMT duplication genes in different organs and stages. [file 1471-2229-11-175-S3.JPEG]
