# Supplementary material for: Phylogenetic analysis and classification of the Brassica rapa SET-domain protein family
Source: BMC Plant Biol. 2011 Dec 14;11:175. doi: 10.1186/1471-2229-11-175 (PMC3264562; doi:10.1186/1471-2229-11-175)
Supplement: Additional file 2 — Table S1. List of ESTs of the B. rapa SET-domain genes. [file 1471-2229-11-175-S2.PDF]

Supplemental Table 1. List of ESTs of the *B. rapa* SET-domain genes.

Gene	EST	Gene	EST
BrKMT1A;1	ES936461, EX023336, EX022109, EX022533, EX077739, EX083048	*BrKMT3;1*	not found
BrKMT1A;2a	not found	*BrKMT3;2*	EX079227
BrKMT1A;2b	not found	*BrKMT3;3*	EX091983, EX047400, EX054864, EE528993
BrKMT1A;2c	not found	*BrKMT3;4a*	EX117440, EX043602, EX126679, EX025721, ES936634
BrKMT1A;3a	EX074417, EX075490, EX074426	*BrKMT3;4b*	EX117440, EX043602, EX126679, EX025721, ES936634
BrKMT1A;3b	EX074417, EX075490, EX074426, EX047370, GR727972, EX127602	*BrKMT3;4c*	EX117440, EX043602, EX126679, EX025721, ES936634
BrKMT1A;4a	EX048884, EX099596, EX058785, EX082059, EX081132	*BrKMT3;4d*	EX117440, EX028686, EX029137, EX126679
BrKMT1A;4b	EX138285, EX139155, ES935237	*BrKMT6A;1*	EX092237, EX061244
BrKMT1A;4c	EX048884, EX099596, EX058785, EX082059, EX081132	*BrKMT6A;2*	ES933610
BrKMT1A;4d	EX048884, EX099596, EX058785, EX082059, EX081132	*BrKMT6A;3a*	not found
BrKMT1A;4e	not found	*BrKMT6A;3b*	not found
BrKMT1A;4f	not found	*BrKMT6B;1a*	not found
BrKMT1A;4g	EX099596, EX082059, EX081132, EX048884, EX058785	*BrKMT6B;1b*	not found
BrKMT1A;4h	not found	*BrKMT6B;2a*	not found
BrKMT1B;1a	EX137551, EX061478, EE529989, ES932215, ES934379	*BrKMT6B;2b*	not found
BrKMT1B;1b	EE529895, ES931860, ES932455, ES933477, ES933589, ES935972	*BrKMT7;1a*	EX048426
BrKMT1B;2a	EX118613, EX053870, EX039238, EX050162, EX058163, EX085689	*BrKMT7;1b*	EX048426
BrKMT1B;2b	EX118613, EX053870, EX039238, EX050162, EX058163, EX085689	*BrKMT7;1c*	EX127951, EX117972, EX023281, EX106289
BrKMT1B;3	EX139382	*BrS-ET;1*	not found
BrKMT1B;4	not found	*BrS-ET;2*	not found
BrKMT2;1a	EX055501	*BrS-ET;3*	EX052815, EX049156, EX046506, EE529014, EX117567
BrKMT2;1b	EX055501, EX090799	*BrS-ET;4a*	EX055451, EX096720, GR727329, EX091740
BrKMT2;2	EX077959	*BrS-ET;4b*	not found
BrKMT2;3a	EX033983, EX124598, EX058254		
BrKMT2;3b	not found		
BrKMT2;3c	EX124598		
