# Supplementary material for: Phylogenetic analysis and classification of the Brassica rapa SET-domain protein family
Source: BMC Plant Biol. 2011 Dec 14;11:175. doi: 10.1186/1471-2229-11-175 (PMC3264562; doi:10.1186/1471-2229-11-175)
Supplement: Additional file 4 — Table S2. List of gene specific primers used in this study. [file 1471-2229-11-175-S4.PDF]

Supplemental Table 2. List of gene specific primers used in this study.

Gene	Forward Primer	Reverse Primer
BrKMT1A;2a	5'-ACGGCTAACCGAAGCTCAAAC-3'	5'-ATGTAGTCATACCCCTCCGGTTC-3'
BrKMT1A;2c	5'-CACAAGCCGGGATTGAATACAC-3'	5'-GTGTTTCTAAGTGTTAGCGCACG-3'
BrKMT1A;4a	5'-TGCATATACCGAAAACGACAG-3'	5'-GGGGACTTACCAGTCTTCCAAC-3'
BrKMT1A;4c	5'-ATGGCTGGCATTGACTATCTTCT-3'	5'-CCTTTCCACTTTTGTATCCCTA-3'
BrKMT1A;4d	5'-GTTTAAGCTTACTCAGCAGCC-3'	5'-AGCCACCGTTGGTTGCATTTG-3'
BrKMT1B;1a	5'- ACTCCAGGTTTACTTTACCCAA-3'	5'-CTTCCTGCTTGTTTTTTAGCAG-3'
BrKMT1B;1b	5'-CAACTTCAGGTTTACTTTACCTCG-3'	5'-CTCTTCCTGCTTGTTTTTTAGCAT-3'
BrKMT1B;2a	5'-CAAACAGTCCCAGCTTGGGC-3'	5'-CTCAAGCGCTGAAATTCCTTCTG-3'
BrKMT1B;2b	5'-GGGAAGTCCCAGTTTAGAAGAG-3'	5'-ATTCAAGTGTTGAAGATTCTTCCA-3'
BrKMT2;2	5'-CCAACTATCAACGGTTATATGCAC-3'	5'-TCCAAAGCTCCAATAAAGTATATGG-3'
BrKMT3';1	5'-GAAATCCAAGTCCAAGTCTGCC-3'	5'-TCTCGAGCTCTCGTCAATTGACTC-3'
BrKMT3';2	5'-AGTTATATCTAAGTCTCTTCCGAGG-3'	5'-CAGAGAGCTTAGTCTGGTTATTATC-3'
BrKMT3';4a	5'-AGATACATACTTGACGAAGAAAATC-3'	5'-CAAAGCCTCTCTTCACAAGTCTT-3'
BrKMT3';4b	5'-CTTAGGAAGCTGAAGCATAAGGTG-3'	5'-CGTTAGGTTCACATCTTCATCCTT-3'
BrKMT3';4c	5'-TGTACACTTGACGGAGAAAACA-3'	5'-ATAGCCTTTGTGCACAAGTCAC-3'
BrKMT3';4d	5'-TTTCTACTTGTGTCAGATAAACTCG-3'	5'-GGTTTCCAGGTCACTGCTTCA-3'
BrKMT6A;3'a	5'-GCTTCCCTTAACCCGGCACAA-3'	5'-GCGTCTTCATACTTTTTCTTGTCAGG-3'
BrKMT6A;3'b	5'-GCTGGAGTAAATAATGATGGAGGG-3'	5'-CTTCATCTTCTTCGTCGTTGCTG-3'
BrKMT6B;1a	5'-GGAGCTATGTAGAGCTATGTATAGACG-3'	5'-ATAATAGAGCCTCTCCCCTTTCGCT-3'
BrKMT6B;1b	5'-TGAGTCAAGAAGAGACAATAAAGCA-3'	5'-CTGTGAAACCTTGAAACTGATCTTC-3'
BrKMT6B;2a	5'-CTGTTCTCAACACCAGATCCCC-3'	5'-GATCTTGGAGCCTTGCCAGAC-3'
BrKMT6B;2b	5'-CTCGTGTTCTAAACACCAGAACCTT-3'	5'-TGCAGATCTTAGAGCCTTGCTATGT-3'
BrKMT7;1a	5'-CTATTGAAAATGCAACATCTCCTAC-3'	5'-CTTCTTTTTGTAATATATATCTTGCG-3'
BrKMT7;1b	5'-TGAGCTTGAAGAGACGAGAAGG-3'	5'-CGCAAGCTGATGACTGAGAAATAC-3'
Actin	5'-TTCAATGTCCCTGCCATGTATGT-3'	5'-TCTTAG CCGTCTCCAGCTCTTGC-3'
